# Metformin decreases progerin expression and alleviates pathological defects of Hutchinson–Gilford progeria syndrome cells

**DOI:** 10.1038/npjamd.2016.26

**Published:** 2016-11-10

**Authors:** Anne-Laure Egesipe, Sophie Blondel, Alessandra Lo Cicero, Anne-Laure Jaskowiak, Claire Navarro, Annachiara De Sandre-Giovannoli, Nicolas Levy, Marc Peschanski, Xavier Nissan

**Affiliations:** 1 INSERM U861, I-STEM, AFM, Institute for Stem cell Therapy and Exploration of Monogenic Diseases, Corbeil Essonnes, France; 2 UEVE, I-STEM, AFM, Institute for Stem cell Therapy and Exploration of Monogenic Diseases, Corbeil Essonnes, France; 3 CECS, I-STEM, AFM, Institute for Stem cell Therapy and Exploration of Monogenic Diseases, Corbeil Essonnes, France; 4 Aix Marseille Université, UMR S 910: Génétique médicale et génomique fonctionnelle, Faculté de médecine Timone, Marseille, France; 5 INSERM, UMR S 910: Génétique médicale et génomique fonctionnelle, Faculté de médecine, Marseille, France

## Abstract

Hutchinson–Gilford progeria syndrome (HGPS) is a rare genetic disorder that causes systemic accelerated aging in children. This syndrome is due to a mutation in the *LMNA* gene that leads to the production of a truncated and toxic form of lamin A called progerin. Because the balance between the A-type lamins is controlled by the RNA-binding protein SRSF1, we have hypothesized that its inhibition may have therapeutic effects for HGPS. For this purpose, we evaluated the antidiabetic drug metformin and demonstrated that 48 h treatment with 5 mmol/l metformin decreases SRSF1 and progerin expression in mesenchymal stem cells derived from HGPS induced pluripotent stem cells (HGPS MSCs). The effect of metformin on progerin was then confirmed in several *in vitro* models of HGPS, i.e., human primary HGPS fibroblasts, *Lmna*^*G609G/G609G*^ mouse fibroblasts and healthy MSCs previously treated with a PMO (phosphorodiamidate morpholino oligonucleotide) that induces progerin. This was accompanied by an improvement in two *in vitro* phenotypes associated with the disease: nuclear shape abnormalities and premature osteoblastic differentiation of HGPS MSCs. Overall, these results suggest a novel approach towards therapeutics for HGPS that can be added to the currently assayed treatments that target other molecular defects associated with the disease.

## Introduction

Lamin A and lamin C are two structural proteins in the nuclear membrane that are expressed the same *LMNA* gene by alternative splicing.^1^ In patients with Hutchinson–Gilford progeria syndrome (HGPS; OMIM #176670), the p.G608G mutation leads to the production of a truncated toxic form of lamin A, called progerin,^2,3^ that accumulates and triggers growth impairment, lipodystrophy, dermal and bone abnormalities, and cardiovascular alterations, leading to a shortened lifespan.^4^ During the past 5 years, several successful strategies have been described for targeting progerin, either by inhibiting its farnesylation with FTIs (farnesyl transferase inhibitors),^5–7^ statins and bisphosphonates,^8^ or by decreasing its production using morpholinos^9–12^ or, more recently, by increasing its degradation, as shown with rapamycin.^13^ Another theoretical approach for the treatment of this disease might be through redirection of the alternative splicing towards the healthy lamin C protein, associated with a parallel decrease in the production of all lamin A isoforms, including progerin.

In 2011, the RNA-binding protein SRSF1 (for serine/arginine-rich splicing factor 1) was shown to affect alternative splicing of *LMNA* in human HGPS primary fibroblasts and mouse *Lmna*^*G609G/G609G*^ fibroblasts.^14^ Pharmacological inhibition of SRSF1 has already been experimentally described as a potential therapeutic approach to modulate alternative splicing of SRSF1-targeted genes through the inhibition of the protein kinase SRPK1.^15–17^ Although several compounds were described, such as SRPIN340, MVRL09 or SPHINX, none of these pharmacological agents is applicable as such for the systemic treatment required for HGPS owing to potential toxicity. This toxicity issue may be resolved by a recent whole-genome transcriptomic analysis that has revealed that SRSF1 expression is transcriptionally regulated by the antidiabetic drug metformin,^18^ which has demonstrated a good safety profile in millions of patients over the past two decades.

We therefore explored the therapeutic potential of metformin for HGPS patients by analyzing its effect on progerin content and HGPS-associated functional defects. For this purpose, we used induced pluripotent stem cells (iPSCs) generated from HGPS patients, which have previously been instrumental as a pharmacological platform for revealing drug effects in earlier studies carried out by our group.^19–21^ In the current study, we show that metformin applied to HGPS cells decreases progerin expression and reduces abnormalities in nuclear shape architecture and premature osteogenic differentiation, suggesting a therapeutic potential for a repurposing of this drug.

## Results

**Figure 1:**
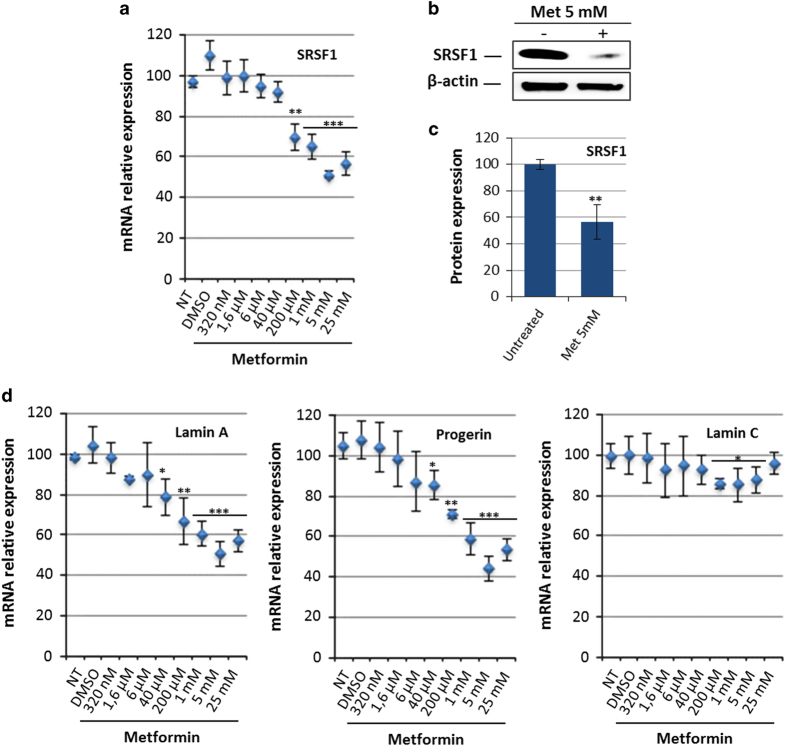
Metformin regulates SRSF1 expression in HGPS MSCs. (**a**) Quantitative PCR analysis of SRSF1 expression in HGPS MSCs treated for 48 h with increasing doses of metformin. Each percentage value represents the mean±s.d. for eight replicates. (**b**) Western blot analysis of SRSF1 expression in HGPS MSCs treated for 48 h with 5 mmol/l (mM) metformin. β-Actin served as a loading control. (**c**) Densitometry measurement of protein levels was relative to untreated cells. The data are presented as mean±s.d. (*n*=3). (**d**) Quantitative PCR analysis of lamin A, progerin and lamin C expression in HGPS MSCs treated with increasing doses of metformin. Each value represents the mean±s.d. of the percentage of eight replicates. Statistical analysis was performed by one-way analysis of variance (ANOVA), using Dunnett’s comparison test. Values of *P*<0.05 were considered significant (*P<0.05, **P<0.01, ***P<0.001). HGPS, Hutchinson–Gilford progeria syndrome; MSC, mesenchymal stem cell; mRNA, messenger RNA.

**Figure 2:**
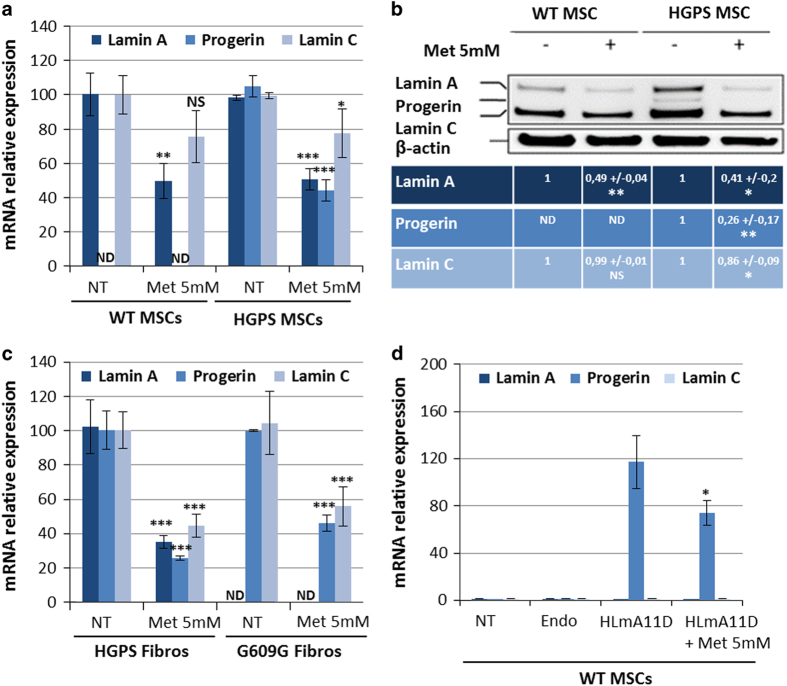
Metformin decreases progerin expression in HGPS MSCs, HGPS fibroblasts, *Lmna^G609G/G609G^* fibroblasts and in healthy MSCs treated with a PMO that induces progerin. (**a**) Quantitative PCR analysis of lamin A, progerin and lamin C expression in wild type (WT) and HGPS MSCs treated for 48 h with 5 mmol/l metformin. Each percentage value represents the mean±s.d. for three replicates. (**b**) Western blot analysis of A-type lamin expression in WT and HGPS MSCs treated with 5 mmol/l metformin. β-Actin served as a loading control. Densitometry measurement of protein levels was relative to untreated cells. The data are presented as mean±s.d. (*n*=3). (**c**) Quantitative PCR analysis of lamin A, progerin and lamin C expression in human HGPS primary fibroblasts (HGPS Fibros) and mouse primary *Lmna^G609G/G609G^* fibroblasts (G609G Fibros) treated with 5 mmol/l metformin. Each percentage value represents the mean ±s.d. for three replicates. (**d**) Quantitative PCR analysis of lamin A, progerin and lamin C expression in WT MSCs treated with HLmnA11D progerin-inducing oligonucleotide in the presence or absence of 5 mmol/l metformin. Each percentage value represents the mean±s.d. for three replicates. Statistical analysis was performed by one-way analysis of variance (ANOVA), using Dunnett’s comparison test. Values of *P*<0.05 were considered significant (NS, *P*>0.05; *P<0.05, **P<0.01, ***P<0.001). HGPS, Hutchinson–Gilford progeria syndrome; Met, metformin; MSC, mesenchymal stem cell; ND, not determined; NS, not significant; PMO, phosphorodiamidate morpholino oligonucleotide.

Initially identified in breast cancer cells (MCF7) by Larsson *et al.*,^18^ the effect of metformin on SRSF1 expression was first evaluated by qPCR, showing a dose-dependent decrease in SRSF1 messenger RNA (mRNA) in HGPS MSCs (Figure 1a). This result was confirmed at the protein level, revealing a decrease in SRSF1 protein of up to 40% after treatment with 5 mmol/l of metformin (Figures 1b and c). Because SRSF1 was reported to regulate the balance between A-type lamins, their expression was then measured 48 h after metformin treatment, showing a correlated dose-dependent decrease in lamin A and progerin expression, with a maximum effect at 5 mmol/l (Figure 1d). All further experiments presented in this study were performed at this dose. The effect of treatment with 5 mmol/l metformin on lamin expression was then confirmed in MSCs derived from non-progeroid iPS cells (wild type; WT MSCs), showing a decrease of 50% in lamin A mRNA expression (Figure 2a). Western blot analysis confirmed the significant decrease in progerin content in HGPS MSCs, as well as the decrease in lamin A and lamin C in WT and HGPS MSCs treated with 5 mmol/l metformin (Figure 2b).

To validate the potential interest of this drug for HGPS, treatment with 5 mmol/l metformin was evaluated in several other cellular models of this disease. Accordingly, similar results were obtained in primary HGPS fibroblasts, confirming that the effect of metformin on lamin expression was not specific to MSCs (Figure 2c). Similarly, *Lmna*^*G609G/G609G*^ mouse primary fibroblasts were treated with 5 mmol/l metformin, showing a decrease in progerin expression of up to 50% under treatment (Figure 2c). Finally, lamins expression were monitored in WT MSCs pre-incubated with a PMO (phosphorodiamidate morpholino oligonucleotide) engineered to induce progerin expression (HLmnA11D),^22^ also resulting in a decrease in progerin through treatment with 5 mmol/l metformin (Figure 2d).

**Figure 3:**
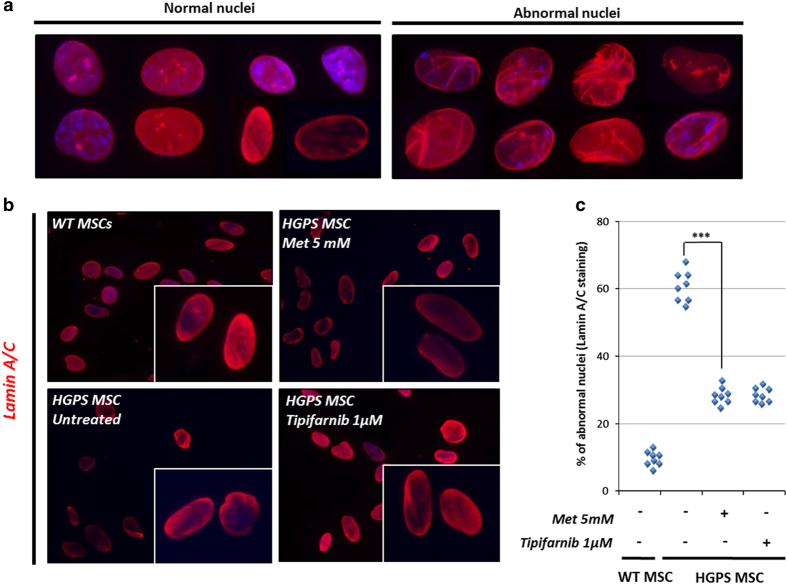
Metformin improves nuclear shape organization in HGPS MSCs. (**a**) Typical nuclear shape organization observed in HGPS MSCs stained for lamin A/C. (**b**) Lamin A/C immunostaining in HGPS MSCs following 48 h of treatment with 5 mmol/l metformin and 1 μmol/l tipifarnib (FTI). (**c**) Quantification of nuclear shape abnormalities following 48 h of treatment with 5 mmol/l metformin and 1 μmol/l tipifarnib (FTI). Statistical analysis was performed by one-way analysis of variance (ANOVA), using Dunnett’s comparison test. Values of *P*<0.05 were considered significant (***P<0.001). HGPS, Hutchinson–Gilford progeria syndrome; FTI, farnesyl transferase inhibitor; Met, metformin; MSC, mesenchymal stem cell.

The therapeutic potential of metformin for HGPS was then explored by measuring its effect on two pathological phenotypes associated with this syndrome, nuclear shape disorganization and premature differentiation. Nuclear shape disorganization in HGPS MSCs was revealed using lamin A/C staining (Figure 3a). After the same number of divisions, HGPS MSCs present approximately 60% of abnormal nuclei, as compared with WT MSCs that present less than 20% of abnormal nuclei (Figures 3b and c). A measure of nuclear shape abnormalities in HGPS MSCs treated with 5 mmol/l metformin revealed a significant rescue of this phenotype at comparable levels to those reached by the reference FTI treatment (1 μmol/l tipifarnib; Figures 3b and c).

**Figure 4:**
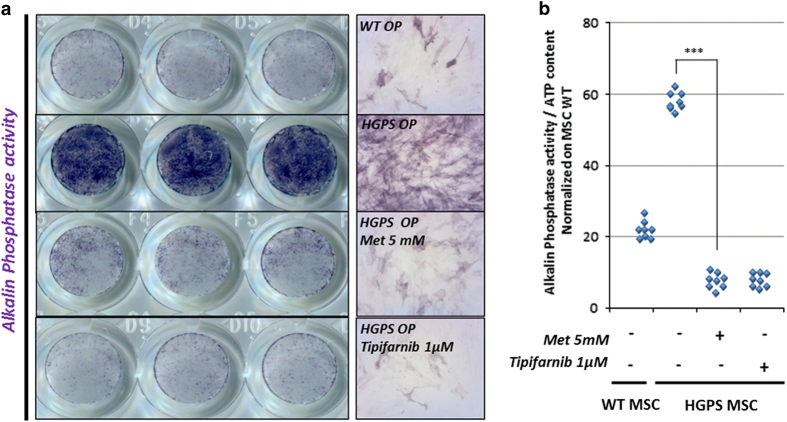
Metformin improves premature osteogenic differentiation of HGPS MSCs. (**a**) Measure of alkaline phosphatase activity in osteogenic progenitors derived from HGPS MSCs (HGPS OP) treated with 5 mmol/l metformin or 1 μmol/l tipifarnib (FTI). (**b**) Quantification of alkaline phosphatase activity in HGPS OP treated with 5 mmol/l metformin or 1 μmol/l tipifarnib (FTI). Statistical analysis was performed by one-way analysis of variance (ANOVA), using Dunnett’s comparison test. Values of *P*<0.05 were considered significant (***P<0.001). HGPS, Hutchinson–Gilford progeria syndrome; FTI, farnesyl transferase inhibitor; Met, metformin; MSC, mesenchymal stem cell.

As previously described, HGPS MSCs are prematurely engaged into osteogenic commitment.^19,21,23^ Osteogenic differentiation of MSCs was monitored through the quantification of alkaline phosphatase (ALP) activity. At this early stage of differentiation, HGPS osteogenic progenitors (HGPS OP) exhibit an increase in ALP activity in comparison to WT OP (Figure 4a). Quantification of ALP activity was performed using spectrophotometry, by measuring the hydrolysis of p-nitrophenyl phosphate into p-nitrophenol, a chromogenic product that absorbs at 405 nm after 4 days of differentiation. Measurement of the effect of 5 mmol/l metformin on this phenotype revealed a significant rescue of ALP activity, at a similar level to that found in cells treated with tipifarnib (Figure 4b).

## Discussion

The main result of this study is the demonstration that the antidiabetic drug metformin reduces progerin expression and alleviates pathological phenotypes of HGPS cells, thus suggesting that it may be interesting to explore its therapeutic potential in patients with progeria.

Since the discovery of the molecular mechanism leading to this syndrome, three different drugs have been repositioned in HGPS for their ability to target progerin toxicity through the inhibition of the protein prenylation process, namely, pravastatin, zoledronate and lonafarnib.^6–8,24,25^ Several alternative approaches have been described more recently that directly target progerin, either by antisense oligonucleotides that mask splicing sites of the mutated pre-mRNAs,^10,11^ by mTOR regulation with rapamycin,^13^ PDGF-BB stimulation^12^ or through retinoids.^26^ Overall, these different strategies have demonstrated that targeting progerin content does indeed improve several pathological phenotypes observed in the cells of patients. In this study, we describe another method for targeting progerin content by repurposing an antidiabetic drug that has shown a very good safety profile since its discovery some decades ago. Even if the exact molecular mechanisms leading to SRSF1 modulation by metformin remain to be explored, especially to understand how this drug acts to regulate the splicing factor expression, this report describes a way of targeting progerin content that may be used alone or in combination with other drugs that target other pathways or pathological molecular mechanisms. Our study reveals that metformin treatment results in a variable, but still significant, decrease in lamin C expression across the cell types. However, the analysis of ratio lamin A/lamin C and progerin/lamin C reveals a very similar effect of metformin across the cell types (Supplementary Figure) suggesting that this drug could act on both transcription and splicing processes.

Taking into account the suggested potential of metformin as an anti-aging drug, its effect on progerin expression may be interesting to explore. Over the past 30 years, several *in vivo* studies have indeed described an increased longevity under metformin treatment. Metformin supplementation (50 mmol/l dose) was shown to increase the average lifespan of *Caenorhabditis elegans* by up to 36%.^27,28^ In mice, several reports have revealed that long-term metformin treatment increases the average lifespan and decreases tumor incidence.^29–31^ In humans, a recent follow-up of patients with type 2 diabetes and atherosclerosis over 20 years has observed all-cause mortality that is 24% lower under metformin treatment.^32^ Another retrospective study of 16,417 patients with type 2 diabetes has indicated a 31% decrease in heart failure in people treated with metformin, as compared with patients treated with other drugs.^33^ More recently, Bannister *et al*.^34^ revealed that among 200,000 subjects, patients with type 2 diabetes initiated with metformin exhibited longer survival than those treated with sulphonylurea and overall longer survival than nondiabetic controls. Even though the possibility that the direct effect of metformin on hyperglycemia and hyperinsulinemia contributes towards these results cannot be excluded, the precise mechanisms of this protective effect remain poorly understood. A link between aging and the appearance of progerin in cells has been repeatedly established over recent years.^35–37^ Therefore, it may be interesting to reconsider the molecular basis of the effects of metformin on aging, taking into account the results of the present study and the possibility that some of the positive outcomes of the drug treatment may arise from an overall decrease in progerin content in aging cells.

## Materials and methods

### Fibroblast culture and reprogramming

The fibroblasts used in this study were isolated from patient biopsies performed in the Assistance Publique Hôpitaux de Marseille for the patient 13-8243 and provided by Coriell Cell Repository (Camden, NJ, USA) for patients AG11513 and GM1972 and controls DM4603 and AG8469. Mouse *Lmna^G609G/G606G^* fibroblasts were extracted from 8-week-old ears, as previously described.^10^ The cultures were maintained in Dulbecco’s modified Eagle’s medium+GlutaMAX II+4,500 mg/l d-glucose (Thermo Fisher Scientific, Waltham, MA, USA), supplemented with 20% fetal bovine serum, research grade (Sigma, St Louis, MO, USA), and 1% sodium pyruvate 100 mmol/l (Thermo Fisher Scientific). The 13-8243 and DM4603 fibroblasts were reprogrammed into iPS cells using Yamanaka’s original method with OCT4, KLF4, SOX2, c-Myc, then transferred using retroviral vectors.^38^ Quality control on the pluripotency and self-renewal capacities of these iPS cells has been published previously by our group.^39^

### Pluripotent stem cell culture and differentiation

The WT and HGPS iPS cells were grown in colonies on mouse embryonic fibroblasts, inactivated with 10 mg/ml mitomycin C seeded at 30,000/cm^2^ and grown as previously described.^39^ For differentiation, iPSCs were differentiated into MSCs using directed protocols for differentiation previously published by our group.^39^

### Cell culture and drug treatments

The MSCs derived from HGPS iPS cells (HGPS MSCs) and WT iPS cells (WT MSCs) were cultured in KnockOut Dulbecco’s modified Eagle’s medium (Invitrogen, Carlsbad, CA, USA), supplemented with 20% fetal bovine serum, research grade (Sigma), 1% nonessential amino acids (Invitrogen), 1% glutamax (Invitrogen) and 0.1% β-mercaptoethanol (Invitrogen). Six hours after seeding, the MSCs were treated with 0.1% dimethyl sulfoxide, 1 μmol/l FTI (tipifarnib, R115777; Selleck Chemicals, Houston, TX, USA) or different concentrations of metformin (1,1-dimethylbiguanide hydrochloride, Sigma). Metformin treatment was repeated 24 h after the initial treatment. The cells were analyzed after 48 h of treatment.

### PMO treatments

The PMOs were obtained from Gene- Tools, LLC (Philomath, OR, USA). The PMOs were transfected according to the manufacturer’s instructions. Briefly, the cells were plated at a high density (80%) and the PMOs were added to the cell cultures at the final concentration of 10 μmol/l, in the presence of endoporters at 6 μmol/l. The sequence used to induce progerin expression in this study was as follows: HLmnA11D (+2 −23)=(5′- GAGACAAAGCAGAGACAACTCACCT-3′).

### Osteogenic differentiation

The cells were seeded at 12,000 cells per well for WT MSCs and 19,000 cells per well for HGPS MSCs in 24-well plates in MSC culture medium and treated as previously described. After 72 h, the MSC medium was replaced by STEMPRO osteogenic induction medium (Invitrogen) in the presence or absence of the different drugs. After 7 days of treatment, the cells were fixed with ethanol 95% and stained by adding either a colorimetric substrate of alkaline phosphatase, 5-bromo-4-chloro-3-indolyl phosphate/nitro blue tetrazolium (Sigma-Aldrich, St Louis, MO, USA), or a chromogenic substrate of this enzyme (absorbance at 405 nm), p-nitrophenyl phosphate (Pierce Biotechnology, Rockford, IL, USA). The cell viability was measured using a luminescent assay, CellTiter-Glo Luminescent Cell Viability Assay (Promega, Madison, WI, USA), based on ATP quantification. The reagent was added to the cell medium and, after 30 min of incubation, the luminescent signal was quantified using an Analyst GT counter luminometer (Molecular Devices, Sunnyvale, CA, USA).

### Quantitative PCR

Total RNA was isolated using an RNeasy Micro extraction kit (Qiagen, Courtaboeuf, France) according to the manufacturer’s protocol. An on-column DNase I digestion was performed to avoid genomic DNA amplification. The RNA level and quality were checked using the Nanodrop technology. A total of 500 ng of RNA was used for reverse transcription using the Superscript III reverse transcription kit (Invitrogen). The quantitative PCR analysis was performed using an ABI 7900 system or a QuantStudio 12 K Flex real-time PCR system (Thermo Fisher Scientific) and TaqMan gene expression Master Mix (Roche, Indianapolis, IN, USA) or Luminaris Probe qPCR Master Mix (Thermo Fisher Scientific), respectively, following the manufacturers’ instructions. Quantification of gene expression was based on the DeltaCt Method and normalized on 18S expression. The PCR primers have been described previously by S. Rodriguez and colleagues.^40^ The primer sequences were lamin A (exons 11/12), 5′- TCTTCTGCCTCCAGTGTCACG-3′ and 5′- AGTTCTGGGGGCTCTGGGT-3′; lamin C (exons 9/10), 5′- CAACTCCACTGGGGAAGAAGTG-3′ and 5′- CGGCGGCTACCACTCAC-3′ and progerin (exons 11/12), 5′- ACTGCAGCAGCTCGGGG-3′ and 5′- TCTGGGGGCTCTGGGC-3′. Taqman MGB probe sequences were lamin A (exon 11), 5′- ACTCGCAGCTACCG-3′; lamin C (exon 10), 5′- ATGCGCAAGCTGGTG-3′ and progerin (exon 11), 5′- CGCTGAGTACAACCT-3′. Reporter and quencher dyes for the *LMNA* locus assays were 5′-6FAM and 3′-non-fluorescent quencher dye (Applied Biosystems). SRSF1 (Assay Hs00199471_m1), SRSF6 (Assay Hs00740177_g1) and 18s (Assay HS_99999901_s1) probes and the primers were provided by Life Technologies.

### Western immunoblotting

Whole-cell lysates of MSC were collected, separated by SDS polyacrylamide gel electrophoresis, and transferred onto polyvinylidene fluoride membrane using the liquid transfer method. The blots were blocked in 10% skim milk (Bio-Rad, Hercules, CA, USA) in Tween 0.1% tris-buffered saline 1× 1 h at room temperature. The primary antibodies were a mouse anti-lamin A/C 1:200 (Millipore, Billerica, MA, USA; JOL2, MAB3211), a mouse anti-SFRS1/SF2 1:500 (LSBio, LS-B2340, Seattle, WA, USA) and a β-actin 1/200,000 (Sigma). The membranes were incubated during the night at 4 °C. Antigen–antibody binding was detected using horseradish peroxidase-conjugated species-specific secondary antibodies (GE-Healthcare, Little Chalfont, UK), followed by enhanced chemiluminescence western blotting detection reagents (Perkin-Elmer, Waltham, MA, USA). The western blot results were quantified using ImageJ software.

### Immunocytochemistry

The cells were fixed in 4% paraformaldehyde (15 min, room temperature) before permeabilization in phosphate-buffered saline supplemented with 0.1% Triton X-100 (Sigma; 5 min, room temperature). They were then blocked for 30 min at room temperature using phosphate-buffered saline with 1% BSA (Sigma-Aldrich). The primary antibodies were incubated for 1 h at room temperature in blocking buffer. Mouse anti-lamin A/C (1:200, clone JOL2, Millipore) was used to detect nuclear shape disorganization. The cells were stained with the species-specific fluorophore-conjugated secondary antibody (Invitrogen; 1 h, room temperature) and the nuclei were visualized using Hoechst 33342 (Invitrogen). The percentage of abnormal nuclei was calculated on the basis of the manual counts carried out on 200 cells for each of the eight replicates.

### Statistical analysis

Statistical analysis was performed by one-way analysis of variance, using Dunnett’s comparison test. The values of *P*<0.05 were considered significant (*P<0.05, **P<0.01, ***P<0.001).

## References

[bib1] Lin, F. & Worman, H. J. Structural organization of the human gene encoding nuclear lamin A and nuclear lamin C. J. Biol. Chem. 268, 16321–16326 (1993). 8344919

[bib2] De Sandre-Giovannoli, A. et al. Lamin a truncation in Hutchinson-Gilford progeria. Science 300, 2055 (2003). 12702809 10.1126/science.1084125

[bib3] Eriksson, M. et al. Recurrent *de novo* point mutations in lamin A cause Hutchinson-Gilford progeria syndrome. Nature 423, 293–298 (2003). 12714972 10.1038/nature01629 PMC10540076

[bib4] Merideth, M. A. et al. Phenotype and course of Hutchinson-Gilford progeria syndrome. N. Engl. J. Med. 358, 592–604 (2008). 18256394 10.1056/NEJMoa0706898 PMC2940940

[bib5] Toth, J. I. et al. Blocking protein farnesyltransferase improves nuclear shape in fibroblasts from humans with progeroid syndromes. Proc. Natl Acad. Sci. USA 102, 12873–12878 (2005). 16129834 10.1073/pnas.0505767102 PMC1193538

[bib6] Gordon, L. B. et al. Clinical trial of a farnesyltransferase inhibitor in children with Hutchinson-Gilford progeria syndrome. Proc. Natl Acad. Sci. USA 109, 16666–16671 (2012). 23012407 10.1073/pnas.1202529109 PMC3478615

[bib7] Gordon, L. B. et al. Impact of farnesylation inhibitors on survival in Hutchinson-Gilford progeria syndrome. Circulation 130, 27–34 (2014). 24795390 10.1161/CIRCULATIONAHA.113.008285 PMC4082404

[bib8] Varela, I. et al. Combined treatment with statins and aminobisphosphonates extends longevity in a mouse model of human premature aging. Nat. Med. 14, 767–772 (2008). 18587406 10.1038/nm1786

[bib9] Scaffidi, P. & Misteli, T. Reversal of the cellular phenotype in the premature aging disease Hutchinson-Gilford progeria syndrome. Nat. Med. 11, 440–445 (2005). 15750600 10.1038/nm1204 PMC1351119

[bib10] Osorio, F. G. et al. Splicing-directed therapy in a new mouse model of human accelerated aging. Sci. Transl. Med. 3, 106ra107 (2011). 10.1126/scitranslmed.3002847 22030750

[bib11] Lee, J. M. et al. Modulation of LMNA splicing as a strategy to treat prelamin A diseases. J. Clin. Invest. 126, 1592–1602 (2016). 26999604 10.1172/JCI85908 PMC4811112

[bib12] Vautrot, V. et al. Enhanced SRSF5 protein expression reinforces lamin A mRNA production in HeLa cells and fibroblasts of progeria patients. Hum. Mutat. 37, 280–291 (2016). 26670336 10.1002/humu.22945

[bib13] Cao, K. et al. Rapamycin reverses cellular phenotypes and enhances mutant protein clearance in Hutchinson-Gilford progeria syndrome cells. Sci. Transl. Med. 3, 89ra58 (2011). 10.1126/scitranslmed.3002346 21715679

[bib14] Lopez-Mejia, I. C. et al. A conserved splicing mechanism of the LMNA gene controls premature aging. Hum. Mol. Genet. 20, 4540–4555 (2011). 21875900 10.1093/hmg/ddr385

[bib15] Amin, E. M. et al. WT1 mutants reveal SRPK1 to be a downstream angiogenesis target by altering VEGF splicing. Cancer Cell 20, 768–780 (2011). 22172722 10.1016/j.ccr.2011.10.016 PMC3574979

[bib16] Gammons, M. V. et al. Targeting SRPK1 to control VEGF-mediated tumour angiogenesis in metastatic melanoma. Br. J. Cancer 111, 477–485 (2014). 25010863 10.1038/bjc.2014.342 PMC4119992

[bib17] Gammons, M. V. et al. Topical antiangiogenic SRPK1 inhibitors reduce choroidal neovascularization in rodent models of exudative AMD. Invest. Ophthalmol. Vis. Sci. 54, 6052–6062 (2013). 23887803 10.1167/iovs.13-12422 PMC3771558

[bib18] Larsson, O. et al. Distinct perturbation of the translatome by the antidiabetic drug metformin. Proc. Natl Acad. Sci. USA 109, 8977–8982 (2012). 22611195 10.1073/pnas.1201689109 PMC3384216

[bib19] Blondel, S. et al. Induced pluripotent stem cells reveal functional differences between drugs currently investigated in patients with hutchinson-gilford progeria syndrome. Stem Cells Transl. Med. 3, 510–519 (2014). 24598781 10.5966/sctm.2013-0168 PMC3973719

[bib20] Lo Cicero, A. & Nissan, X. Pluripotent stem cells to model Hutchinson-Gilford progeria syndrome (HGPS): current trends and future perspectives for drug discovery. Ageing Res. Rev. 24, 343–348 (2015). 26474742 10.1016/j.arr.2015.10.002

[bib21] Blondel, S. et al. Drug screening on Hutchinson Gilford progeria pluripotent stem cells reveals aminopyrimidines as new modulators of farnesylation. Cell Death Dis. 7, e2105 (2016). 26890144 10.1038/cddis.2015.374 PMC5399184

[bib22] Luo, Y. B. et al. Antisense oligonucleotide induction of progerin in human myogenic cells. PLoS ONE 9, e98306 (2014). 24892300 10.1371/journal.pone.0098306 PMC4044034

[bib23] Scaffidi, P. & Misteli, T. Lamin A-dependent misregulation of adult stem cells associated with accelerated ageing. Nat. Cell Biol. 10, 452–459 (2008). 18311132 10.1038/ncb1708 PMC2396576

[bib24] Yang, S. H. et al. Blocking protein farnesyltransferase improves nuclear blebbing in mouse fibroblasts with a targeted Hutchinson-Gilford progeria syndrome mutation. Proc. Natl Acad. Sci. USA 102, 10291–10296 (2005). 16014412 10.1073/pnas.0504641102 PMC1174929

[bib25] Yang, S. H. et al. Assessing the efficacy of protein farnesyltransferase inhibitors in mouse models of progeria. J. Lipid Res. 51, 400–405 (2010). 19965595 10.1194/jlr.M002808 PMC2803242

[bib26] Kubben N., Brimacombe K. R., Donegan M., Li Z. & Misteli T. A high-content imaging-based screening pipeline for the systematic identification of anti-progeroid compounds. Methods 96, 46–58 (2016). 26341717 10.1016/j.ymeth.2015.08.024 PMC6317068

[bib27] Onken, B. & Driscoll, M. Metformin induces a dietary restriction-like state and the oxidative stress response to extend *C. elegans* Healthspan via AMPK, LKB1, and SKN-1. PLoS ONE 5, e8758 (2010). 20090912 10.1371/journal.pone.0008758 PMC2807458

[bib28] Cabreiro, F. et al. Metformin retards aging in *C. elegans* by altering microbial folate and methionine metabolism. Cell 153, 228–239 (2013). 23540700 10.1016/j.cell.2013.02.035 PMC3898468

[bib29] Dilman, V. M. & Anisimov, V. N. Effect of treatment with phenformin, diphenylhydantoin or L-dopa on life span and tumour incidence in C3H/Sn mice. Gerontology 26, 241–246 (1980). 7390164 10.1159/000212423

[bib30] Anisimov, V. N. et al. Metformin decelerates aging and development of mammary tumors in HER-2/neu transgenic mice. Bull. Exp. Biol. Med. 139, 721–723 (2005). 16224592 10.1007/s10517-005-0389-9

[bib31] Anisimov, V. N. et al. Metformin slows down aging and extends life span of female SHR mice. Cell Cycle 7, 2769–2773 (2008). 18728386 10.4161/cc.7.17.6625

[bib32] Roussel, R. et al. Metformin use and mortality among patients with diabetes and atherothrombosis. Arch. Intern. Med. 170, 1892–1899 (2010). 21098347 10.1001/archinternmed.2010.409

[bib33] Masoudi, F. A. et al. Trends in acute myocardial infarction in 4 US states between 1992 and 2001: clinical characteristics, quality of care, and outcomes. Circulation 114, 2806–2814 (2006). 17145994 10.1161/CIRCULATIONAHA.106.611707

[bib34] Bannister, C. A. et al. Can people with type 2 diabetes live longer than those without? A comparison of mortality in people initiated with metformin or sulphonylurea monotherapy and matched, non-diabetic controls. Diabetes Obes. Metab. 16, 1165–1173 (2014). 25041462 10.1111/dom.12354

[bib35] Scaffidi, P. & Misteli, T. Lamin A-dependent nuclear defects in human aging. Science 312, 1059–1063 (2006). 16645051 10.1126/science.1127168 PMC1855250

[bib36] Cao, K. et al. Progerin and telomere dysfunction collaborate to trigger cellular senescence in normal human fibroblasts. J. Clin. Invest. 121, 2833–2844 (2011). 21670498 10.1172/JCI43578 PMC3223819

[bib37] McClintock, D. et al. The mutant form of lamin A that causes Hutchinson-Gilford progeria is a biomarker of cellular aging in human skin. PLoS ONE 2, e1269 (2007). 18060063 10.1371/journal.pone.0001269 PMC2092390

[bib38] Takahashi, K. et al. Induction of pluripotent stem cells from adult human fibroblasts by defined factors. Cell 131, 861–872 (2007). 18035408 10.1016/j.cell.2007.11.019

[bib39] Nissan, X. et al. Unique preservation of neural cells in Hutchinson-Gilford progeria syndrome is due to the expression of the neural-specific miR-9 microRNA. Cell Rep. 2, 1–9 (2012). 22840390 10.1016/j.celrep.2012.05.015

[bib40] Rodriguez, S., Coppedè, F., Sagelius, H.&Eriksson, M. Increased expression of the Hutchinson-Gilford progeria syndrome truncated lamin A transcript during cell aging. Eur. J. Hum. Genet. 17, 928–937 (2009). 19172989 10.1038/ejhg.2008.270 PMC2986496

