# Supplementary figures and images for: Metformin decreases progerin expression and alleviates pathological defects of Hutchinson–Gilford progeria syndrome cells
Source: NPJ Aging Mech Dis. 2016 Nov 10;2:16026–. doi: 10.1038/npjamd.2016.26 (PMC5515002; doi:10.1038/npjamd.2016.26)

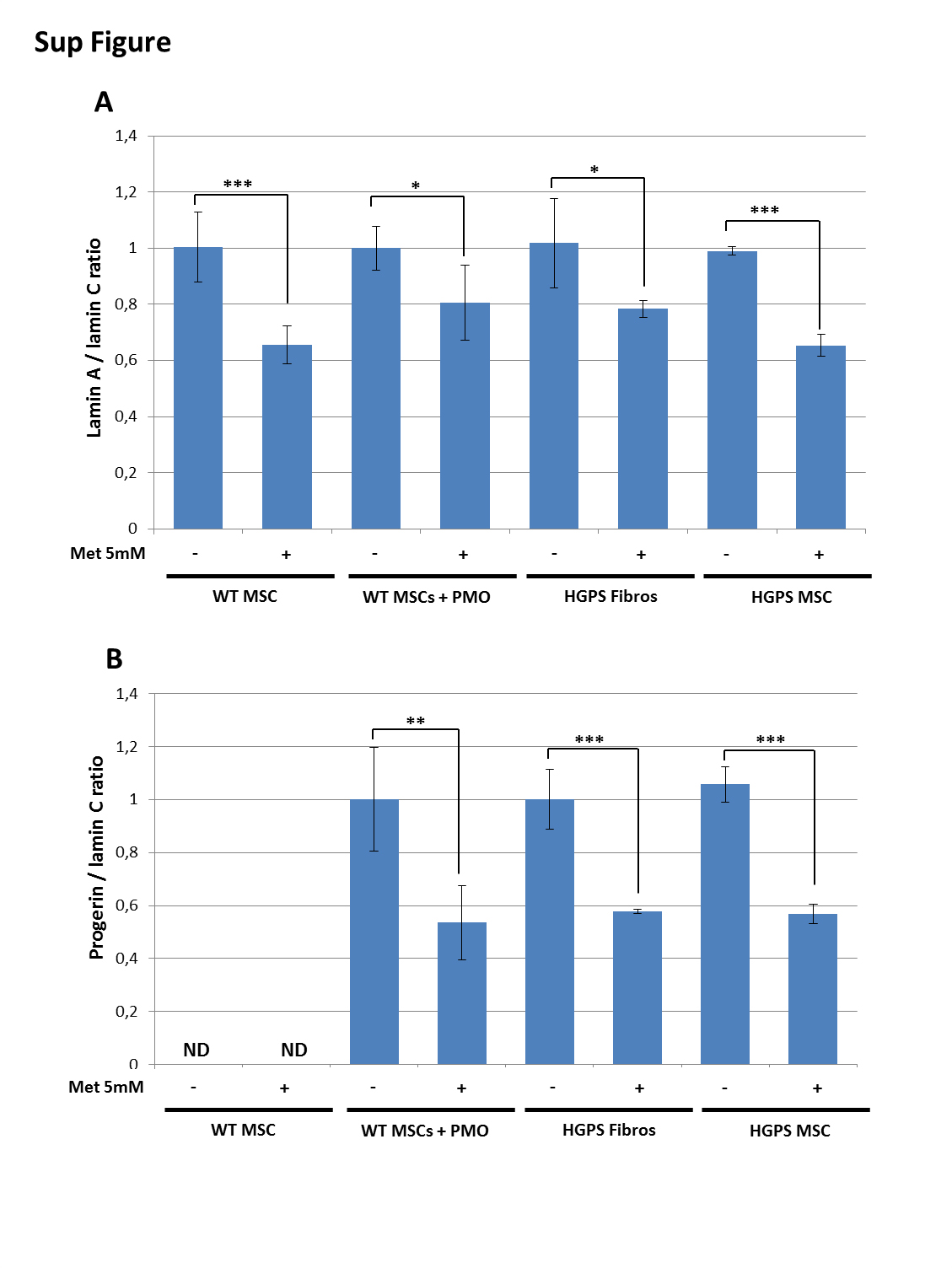

Supplement: Supplementary Figure [file npjamd201626-s1.jpg]
